# Supplementary material for: The Evolution and Characterization of the RNA Interference Pathways in Lophotrochozoa
Source: Genome Biol Evol. 2024 May 7;16(5):evae098. doi: 10.1093/gbe/evae098 (PMC11114477; doi:10.1093/gbe/evae098)
Supplement: evae098_Supplementary_Data [file evae098_supplementary_data.zip › Supplementary_materials.zip!/selected_miRNAs/NoGe_selected_miRNAs/mirdeep_pdf_files/NMRB01000030.1_Notospermus_geniculatus_strain_Ushimado_scaffold30,_whole_genome_shotgun_sequence_1262.pdf]

Provisional ID	:	NMRB01000030.1_Notospermus_geniculatus_strain_Ushimado_scaffold30,_whole_genome_shotgun_sequence_1262
Score total	:	1738
Score for star read(s)	:	3.9
Score for read counts	:	1732.5
Score for mfe	:	2.2
Score for randfold	:	0
Score for cons. seed	:	-0.6
Total read count	:	3410
Mature read count	:	2408
Loop read count	:	0
Star read count	:	1002

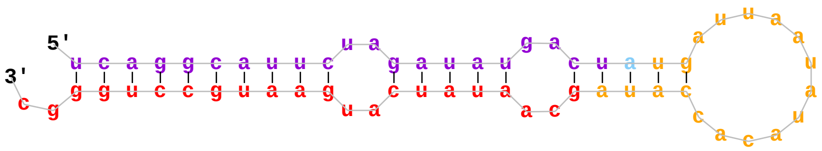

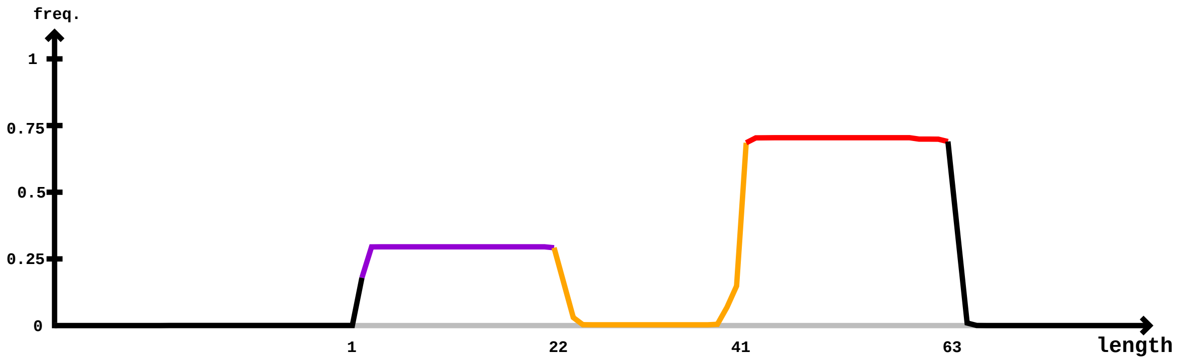


```
                                   Star                                  Mature
5'- aucaagaagacagaucaucuggccugggugucaggcauucuagauaugacuaugauuaauauacaccauagcaauaucaugaaugccugggcauccagguccagguuucuca   -3'    obs
    aucaagaagacagaucaucuggccugggugucaggcauucuagauaugacuaugauuaauauacaccauagcaauaucaugaaugccugggcauccagguccagguuucuca          exp
    ...........(((..((((((((((((((((((((((((..(((((..(((((............)))))..)))))..)))))))).)))))))))).)))))).)))..   reads   mm   sample
    ..............................ucaggcauucuagauaugac..............................................................   1       0    Mm3
    ..............................ucaggcauucuagauaugacu.............................................................   33      0    Mm3
    ..............................ucaggcauucuagauaGgacu.............................................................   1       1    Mm3
    ..............................ucaggcauucuagauauCacu.............................................................   1       1    Mm3
    ..............................ucGggcauucuagauaugacu.............................................................   2       1    Mm3
    ..............................ucaggcauucuagauaugacG.............................................................   1       1    Mm3
    ...............................cagAcauucuagauaugacu.............................................................   1       1    Mm3
    ...............................caggcauucuagauaugacu.............................................................   7       0    Mm3
    ...............................caggcauucuagauaugacua............................................................   1       0    Mm3
    ....................................................................uagcaauaucaugaaugccuggg.....................   3       0    Mm3
    ....................................................................uagcaauaucaugaaugccugggc....................   35      0    Mm3
    .....................................................................agcaauaucaugaaugccugggc....................   47      0    Mm3
    .....................................................................agcaauaucaugaaugcAugggc....................   2       1    Mm3
    .....................................................................agcaauaucaugaaugccugggcU...................   1       1    Mm3
    ......................................................................Ncaauaucaugaaugccugggc....................   1       1    Mm3
    ......................................................................gcaauaucaugaaugccugggU....................   1       1    Mm3
    ......................................................................gcaauaucGugaaugccugggc....................   1       1    Mm3
    ......................................................................gcaauaucauUaaugccugggc....................   1       1    Mm3
    ......................................................................gcaauaucaugaaugcAugggc....................   1       1    Mm3
    ......................................................................gcaauaucaugaaugccGgggc....................   1       1    Mm3
    ......................................................................gcaauaucaugaaugccugggc....................   242     0    Mm3
    ......................................................................gcaauaucaugaaugcUugggc....................   1       1    Mm3
    ......................................................................gcaauaucaugaGugccugggc....................   1       1    Mm3
    ......................................................................gcaauaucaugaaugccAgggc....................   4       1    Mm3
    ......................................................................gcaauaucaugaaugccugggca...................   4       0    Mm3
    ......................................................................gcaauaucaugaaugcUugggca...................   1       1    Mm3
    ......................................................................gcaauaucaugaaugccugggcU...................   2       1    Mm3
    ......................................................................gcaauaucaugaaugccugggcUu..................   1       1    Mm3
    .......................................................................caauaucaugaaugccugggc....................   4       0    Mm3
    .......................................................................caauaucaugaaugccugggca...................   7       0    Mm3
    .......................................................................caauaucaugaaugccugggcU...................   5       1    Mm3

    ..............................ucaggcauucuagauaugac..............................................................   6       0    Fm3
```

```
                              Star                                      Mature
aucaagaagacagaucaucuggccugggugucaggcauucuagauaugacuaugauuaauauacaccauagcaauaucaugaaugccugggcauccagguccagguuucuca
..............................ucaggcauucGagauaugacu.............................................................   1     1   Fm3
..............................ucaggcauuUuagauaugacu.............................................................   1     1   Fm3
..............................ucaggcauucuagauaugacu.............................................................   222   0   Fm3
..............................ucGggcauucuagauaugacu.............................................................   2     1   Fm3
..............................ucaggcauucuagauauUacu.............................................................   1     1   Fm3
..............................ucaggcauucuagauauCacu.............................................................   1     1   Fm3
..............................ucaggcauucuagauaCgacu.............................................................   4     1   Fm3
..............................ucaggcauucuagauauAacu.............................................................   5     1   Fm3
..............................ucaggcauucuagauaugacua............................................................   2     0   Fm3
...............................caggcauucuUgauaugacu.............................................................   1     1   Fm3
...............................caggcauucuagauaugGcu.............................................................   1     1   Fm3
...............................caggcauucuagauaugacu.............................................................   71    0   Fm3
...............................caggcauucuagauaugacuau...........................................................   1     0   Fm3
...................................................................auagcaauaucaugaaugccu........................   6     0   Fm3
....................................................................uagcaauaucaugaaugccu........................   10    0   Fm3
....................................................................uagcaauaucaugaaugccuggg.....................   3     0   Fm3
....................................................................uagcaauaucaugaaugccugggc....................   9     0   Fm3
....................................................................Cagcaauaucaugaaugccugggc....................   1     1   Fm3
.....................................................................agcaauaucaugaaugccugggc....................   26    0   Fm3
.....................................................................agcaauaucaugaaugccugggA....................   1     1   Fm3
......................................................................gcaauaucaugaaugccu........................   2     0   Fm3
......................................................................gcaauaucaugaaugccGgggc....................   1     1   Fm3
......................................................................gcaauaucaugaaugccugggc....................   150   0   Fm3
......................................................................gcaauaucaugaaugccuUggc....................   1     1   Fm3
......................................................................gcaauaucaugaaugUcugggc....................   1     1   Fm3
......................................................................Ucaauaucaugaaugccugggca...................   1     1   Fm3
......................................................................gcaauaucaugaaugccugggca...................   2     0   Fm3
.......................................................................caauaucaugaaugccugggcU...................   2     1   Fm3
.......................................................................caauaucaugaaugccugggca...................   4     0   Fm3
........................................................................aauaucaugaaugccugggc....................   1     0   Fm3

..........cagaucaucuggccugggug..................................................................................   1     0   Mm1
..............................ucGggcauucuagauaugacu.............................................................   4     1   Mm1
.....................................................................agcaauaucaugaaugccugggc....................   1     0   Mm1
......................................................................gcaauaucaugaaugccugggc....................   16    0   Mm1
.......................................................................caauaucaugaaugccugggc....................   2     0   Mm1
.......................................................................caauaucaugaaugccuCggc....................   1     1   Mm1

..............................ucaggcauucuagauaugac..............................................................   4     0   Mm2
..............................ucaggcauucuagauaugacu.............................................................   35    0   Mm2
..............................ucGggcauucuagauaugacu.............................................................   1     1   Mm2
..............................ucaUgcauucuagauaugacu.............................................................   1     1   Mm2
..............................ucaggcauucuagauaugacua............................................................   2     0   Mm2
...............................caggcauucuagauaugacu.............................................................   16    0   Mm2
....................................................................uagcaauaucaugaaugccugggc....................   24    0   Mm2
....................................................................uagcaauaucaugaaugGcugggc....................   1     1   Mm2
....................................................................uagcaauaucaugaaugccugggU....................   1     1   Mm2
....................................................................uagcaauaucaugaaugUcugggc....................   1     1   Mm2
.....................................................................agcaauaucaugaaugccuggg.....................   2     0   Mm2
.....................................................................agcaauaucaugaaugccugggc....................   34    0   Mm2
.....................................................................agcaauaucaugaaugAcugggc....................   1     1   Mm2
.....................................................................agcaauaucaugaaugccugggca...................   2     0   Mm2
.....................................................................agcaauaucaugaaugccugggcUu..................   1     1   Mm2
......................................................................gcaauaucaugaaugccugU......................   1     1   Mm2
......................................................................gcaauaucaugaaugcAugggc....................   3     1   Mm2
......................................................................gcaaAaucaugaaugccugggc....................   1     1   Mm2
......................................................................gcaauaucaugaaugccugggc....................   290   0   Mm2
......................................................................gcaauaucaugaaugcGugggc....................   1     1   Mm2
......................................................................gcaauNucaugaaugccugggc....................   1     1   Mm2
......................................................................Ucaauaucaugaaugccugggc....................   1     1   Mm2
......................................................................gcaauaucaugaaugccAgggc....................   1     1   Mm2
......................................................................gcaauaucaugaaugccGgggc....................   3     1   Mm2
......................................................................gcaauGucaugaaugccugggc....................   3     1   Mm2
......................................................................gcaauaucaugaaugccugggca...................   3     0   Mm2
......................................................................gcaauaucaugaaugccugggcU...................   3     1   Mm2
......................................................................gcaauaucaugaaugccugggcG...................   1     1   Mm2
......................................................................gcaauaucaugaaugccugggcUu..................   4     1   Mm2
.......................................................................caauaucaugaaugccugggc....................   3     0   Mm2
.......................................................................caauaucaugaaugccugggca...................   6     0   Mm2
.......................................................................caauaucaugaaugccugggcU...................   3     1   Mm2
```

```
                             Star                                      Mature
aucaagaagacagaucaucuggccugggugucaggcauucuagauaugacuaugauuaauauacaccauagcaauaucaugaaugccugggcauccagguccagguuucuca
.......................................................................caauaucaugaaugccugggcau..................   1     0    Mm2
.......................................................................caauaucaugaaugccugggcauA.................   1     1    Mm2
.........................................................................auaucaugaaugccugggc....................   1     0    Mm2

..............................ucaggcauucuagauaugacu.............................................................   50    0    Fm2
..............................ucaggcauucuagauauUacu.............................................................   1     1    Fm2
..............................ucaggcauucuagauaugacua............................................................   8     0    Fm2
..............................ucaggcauucuagauaugacuaA...........................................................   5     1    Fm2
...............................caggcauucuagauaugacu.............................................................   51    0    Fm2
...............................cGggcauucuagauaugacu.............................................................   5     1    Fm2
...............................caggUauucuagauaugacua............................................................   5     1    Fm2
...............................caggcauucuagauaugacuaugauuaauauacaccaua..........................................   7     0    Fm2
....................................................................uagcaauaucaugaaugccuggg.....................   1     0    Fm2
....................................................................uagcaauaucaugaauCccuggg.....................   1     1    Fm2
....................................................................uagcaauaucaugaauAccugggc....................   1     1    Fm2
....................................................................uagcaauaucaugaaugAcugggc....................   2     1    Fm2
....................................................................uagcaauaucaugaaugccugggU....................   6     1    Fm2
....................................................................uagcaauaucaugaaugccugggc....................   52    0    Fm2
....................................................................uagcaauaucaugaaugccugggcU...................   13    1    Fm2
.....................................................................Cgcaauaucaugaaugccugggc....................   8     1    Fm2
.....................................................................agcaauaucaugaaugccugggU....................   1     1    Fm2
.....................................................................agcaauaucaugaaugcAugggc....................   1     1    Fm2
.....................................................................agcaauaucaugaaCgccugggc....................   1     1    Fm2
.....................................................................agcaCuaucaugaaugccugggc....................   1     1    Fm2
.....................................................................agcaauaucaugaaugccugggc....................   90    0    Fm2
......................................................................gcaauaucaugaaugccCgggc....................   2     1    Fm2
......................................................................gcaauaucaugaaugcUugggc....................   2     1    Fm2
......................................................................gcaauaucaugaaugccGgggc....................   2     1    Fm2
......................................................................gcaauGucaugaaugccugggc....................   9     1    Fm2
......................................................................gcaauaucauUaaugccugggc....................   1     1    Fm2
......................................................................gcaauaucaugaaugccugggA....................   1     1    Fm2
......................................................................gcaauaucauCaaugccugggc....................   1     1    Fm2
......................................................................gcaauaucaugaaCgccugggc....................   1     1    Fm2
......................................................................gcaauaucGugaaugccugggc....................   1     1    Fm2
......................................................................gcaauaucaugaaugccAgggc....................   3     1    Fm2
......................................................................gcaauaucaugaaugccugggc....................   698   0    Fm2
......................................................................gcaauaucaugaaugcGugggc....................   2     1    Fm2
......................................................................gcaauaucUugaaugccugggc....................   1     1    Fm2
......................................................................gcaauaucaugaaugcAugggc....................   2     1    Fm2
......................................................................gcaauaucaugaaugccuggUc....................   1     1    Fm2
......................................................................gcaCuaucaugaaugccugggc....................   1     1    Fm2
......................................................................gcaauaucaugaaugccugggca...................   2     0    Fm2
......................................................................gcaauaucaugaaugccugggcUu..................   8     1    Fm2
.......................................................................caauaucaugaaugccugggcUu..................   9     1    Fm2

..............................ucaggcauucuagauaugac..............................................................   1     0    Fm1
..............................ucaggcauucAagauaugacu.............................................................   1     1    Fm1
..............................uGaggcauucuagauaugacu.............................................................   1     1    Fm1
..............................ucaggcauucuagauaugacu.............................................................   179   0    Fm1
..............................ucGggcauucuagauaugacu.............................................................   8     1    Fm1
..............................ucaggcauucuagauaCgacu.............................................................   2     1    Fm1
..............................ucaggcauucuUgauaugacu.............................................................   1     1    Fm1
..............................ucaggcauucuagauaugacG.............................................................   2     1    Fm1
..............................ucaggcauucCagauaugacu.............................................................   1     1    Fm1
..............................ucaggcauucuagauauCacu.............................................................   3     1    Fm1
..............................ucaggcauucuagauaugacua............................................................   16    0    Fm1
..............................ucaggcauucuagauaugacuaA...........................................................   5     1    Fm1
...............................caggcauucuagauauCacu.............................................................   1     1    Fm1
...............................caggcauucuagauaugacG.............................................................   2     1    Fm1
...............................caggcauucuagauaugacu.............................................................   124   0    Fm1
...............................caggcauucuagauaugCcu.............................................................   2     1    Fm1
...............................cGggcauucuagauaugacu.............................................................   2     1    Fm1
...............................caggcauucuaUauaugacu.............................................................   1     1    Fm1
...............................caggcauuUuagauaugacu.............................................................   2     1    Fm1
...............................caggcauucuagauaugacua............................................................   10    0    Fm1
...............................caggcauucuagauaugCcuau...........................................................   1     1    Fm1
...............................caggcauGcuagauaugacuau...........................................................   1     1    Fm1
...............................caggcauucuagauaugacuau...........................................................   74    0    Fm1
...............................caggcauucuagauaugacuaA...........................................................   5     1    Fm1
...............................caggcauucuagauaugacuauU..........................................................   1     1    Fm1
```

Star Mature

```
aucaagaagacagaucaucuggccugggugucaggcauucuagauaugacuaugauuaauauacaccauagcaauaucaugaaugccugggcauccagguccagguuucuca
...............................caggcauucuagauaugacuaugauuaauauacaccau...........................................   3     0    Fm1
................................aggcauucuagauaugacuaA...........................................................   1     1    Fm1
....................................................................uagcaauaucaugaaugccuggg.....................   20    0    Fm1
....................................................................uagcaauaucaugaaugUcugggc....................   1     1    Fm1
....................................................................uagcaauaucaugaaugccuggAc....................   1     1    Fm1
....................................................................uagcaauaucaugaaugccugggc....................   22    0    Fm1
....................................................................uagcaauaucaugaaugccugggU....................   11    1    Fm1
.....................................................................agcaauaucaugaaugcGugggc....................   1     1    Fm1
.....................................................................agcaauaucaugaaAgccugggc....................   1     1    Fm1
.....................................................................agcaauaucaugaaugccugggU....................   2     1    Fm1
.....................................................................agcaauaucaugaaugcUugggc....................   1     1    Fm1
.....................................................................agcaauaucaugaaugcAugggc....................   2     1    Fm1
.....................................................................agcaauaucaugaaugccugggc....................   50    0    Fm1
......................................................................Ncaauaucaugaaugccugggc....................   1     1    Fm1
......................................................................gcGauaucaugaaugccugggc....................   1     1    Fm1
......................................................................gcaauaucaugaaugccAgggc....................   2     1    Fm1
......................................................................gcaauaucaugaaugccGgggc....................   2     1    Fm1
......................................................................gcaauaucaugaaugcAugggc....................   1     1    Fm1
......................................................................gcaauaucaugaaugccugggc....................   321   0    Fm1
......................................................................gcaauaucaugaaugccugggU....................   3     1    Fm1
......................................................................gcaauaucaugaaGgccugggc....................   1     1    Fm1
......................................................................gcaauaucaugaaugccCgggc....................   3     1    Fm1
......................................................................gcaauaucauAaaugccugggc....................   2     1    Fm1
......................................................................gcaauaucaugaaugccugggca...................   9     0    Fm1
......................................................................gcaauaucaugaaugccugggcU...................   3     1    Fm1
......................................................................gcaauaucaugaaugccugggcUu..................   8     1    Fm1
.......................................................................caauaucaugaaugccugggca...................   15    0    Fm1
```
